# The Book World of Medicine and Science

**Published:** 1902-12-20

**Authors:** 


### THE BOOK WORLD OF MEDICINE AND SCIENCE.

Massage and the Original Swedish Movements. Their Application to Various Diseases of the Body. By Kurre W. Ostrom. Fifth edition, revised and enlarged. Pp. 181 and 115 figures. (Philadelphia: P. Blakeston's, Son and Co. 1902. Price $1 net.)

Mechanotherapeutics now rightly occupy a prominent position in the rational treatment of disease. But the successful practice of massage and a beneficial conduct of Swedish and other movements necessitate care, judgment, and experience. The present work is a well-designed and carefully-executed manual, concise in expression, accurate in description, and peculiarly free from most of the objectionable features which only too readily and much too frequently creep into this class of book. The fact that the volume is now in its fifth edition indicates that it has been appreciated. One of its most praiseworthy features is the very excellent, although simple, series of figures which go far to elucidate the text. The section on massage is brief, but sufficient for most needs. The description of the Swedish Movements is also such as to give a clear indication of their characters. The section dealing with the practical application of massage and various movements to different diseases of the body is certainly open to criticism, but the author is well aware of the dangers incident to unregulated use of these agents, and rightly warns his readers that massage and movement treatment should be applied only by educated and properly trained persons, and with due regard to the physician's directions. There is a useful bibliography which will prove of service to serious students of the subject. Although printed and published in America, the work may well find readers on this side "the herring-pond."

The Science and Art of Prescribing. By E. H. Colbeck, M.D., and Arnold Chaplin, M.D. (London: Henry Kimpton. 1902. Price 5s. Pp. 186).

A small and handy book on the above subject has long been urgently required alike by students for the acquisition of the knowledge necessary for the passing of examinations and by practitioners in the active pursuit of their calling, for the teaching of practical pharmacy in our medical schools is admittedly inefficient. The little work which Drs. Colbeck and Chaplin have prepared to fill the hiatus in our medical knowledge caused by this want of system in the teaching provided by our medical schools is in every way a practical guide to the art of prescribing. The opening chapters are concerned with the methods of prescribing, with the administration of drugs, and with the subject of incompatibility. Several very valuable tables are included in this portion of the work on the solubility of drugs in water, alcohol and glycerine, and on the dosage, form of administration and correction of taste of all drugs which are commonly prescribed. The second portion of the book describes the application of the methods of prescribing, and the subject is divided into eight chapters, which deal independently with the treatment of diseases affecting the various systems, such as the alimentary, respiratory, and nervous systems. These chapters are far more than illustrated accounts of the methods employed in prescribing, indeed they savour very strongly of practical therapeutics. The illustrated prescriptions contain among their number some good examples of medicinal formulæ, written and used by some of the most brilliant prescribers of the last century. Some of the formulæ are taken from well-known works on therapeutics and treatment, while others are borrowed from the pharmacopœias of our London hospitals. It will be understood from the above description that "The Art and Science of Prescribing" is not only a book for the instruction of students and others who desire to obtain a complete introduction to the subject, but it is also a practical guide for handy and immediate reference, especially suitable for practitioners. The printing, binding, and general get-up of this little work are all that can be desired; it is sufficiently small to be easily carried and sufficiently comprehensive to afford information in cases of doubt as to treatment and prescribing as a medical man is likely to be confronted with in the execution of his ordinary duties.

Organic Chemistry. By W. H. Perkin, Jun., Ph.D., F.R.S., and F. Stanley Kipping, Ph.D., D.Sc.(Lond.), F.R.S. (London and Edinburgh: W. and R. Chambers. 1902. Price 7s. 6d.)

Organic Chemistry, for its proper comprehension, requires lucid explanation, and this is what it obtains in the book before us. Every work on such a subject must, as a matter of course be full of technicalities and formulæ, and in that respect this is only as other books of like nature. What we find here, however, more than in some treatises that we could name, is the clear conception and the orderly exposition of the phenomena in question, by aid of which the student is enabled to grasp the lesson which it is the object of the writers to teach. From the construction of the book it would appear that it is intended to be read at least twice through. Obviously enough in dealing with so abstruse a subject as organic chemistry the student not only grows in knowledge as he reads but at the same time increases his power of understanding. Thus an author might safely towards the end of the book use arguments and give illustrations which if introduced at the beginning would be bewildering and incomprehensible. Such a method, however, would produce an ill-balanced book, and one quite unfitted to act as a work of reference. To meet this difficulty the authors of this book have made use of two kinds of type, the large type to be mastered at the first reading and both large and small at the second. It is hardly necessary to say that some knowledge of inorganic chemistry, and of the general principles of the science, is presupposed on the part of the reader. That being so, the first part of the work is devoted to general considerations regarding the composition and analysis of organic compounds, the deduction of formulæ from the results of analysis, and the composition or structure of organic compounds; after which, in a series of chapters, the fatty compounds are in turn described. Part II. begins with an account of coal-tar, its treatment and its derivatives; the properties of benzene are described, and then in a series of chapters the aromatic compounds are successively dealt with; after which there are chapters on alkaloids, on dyes, and on optical- and stereo-isomerism. Thus, without entering too deeply into any one phase of the wide range of subjects included in the term organic chemistry, a very practical account of the whole is offered to the student. Finally, as an appendix, an account, which will be specially welcomed by medical students, is given of the constituents of plants and animals. As we have already said, the exposition of both facts and theories is made very elear without losing thereby in accuracy. It is a book which can be highly recommended.

